# Editorial: Plant architectural models and crop production

**DOI:** 10.3389/fpls.2024.1430205

**Published:** 2024-06-04

**Authors:** Véronique Letort, MengZhen Kang, Philippe de Reffye

**Affiliations:** ^1^ Laboratory of Mathematics and Computer Sciences (MICS), CentraleSupelec, Paris-Saclay University, Gif-sur-Yvette, France; ^2^ School of Artificial Intelligence, University of Chinese Academy of Sciences, Beijing, China; ^3^ State Key Laboratory for Management and Control of Complex Systems, Institute of Automation, Chinese Academy of Science (CAS), Beijing, China; ^4^ UMR botAnique et Modélisation de l'Architecture des Plantes et des végétations (AMAP), Montpellier University, Centre de coopération internationale en recherche agronomique pour le développement (CIRAD), Centre National de Recherche Scientifique (CNRS), Institut national de recherche pour l'agriculture, l'alimentation et l'environnement (INRAE), Institut de recherche pour le développement (IRD), Montpellier, France

**Keywords:** functional-structural model, mechanistic model, statistical model, water use 10 efficiency, crop physiology, ExGxM interactions

Deciphering plant growth and its complex interactions with environmental and management influences requires more than mere observations. Paraphrasing Saint-Exupery’s famous citation, we could say, “*It is only with the [model] that one can see rightly; what is essential is invisible to the eye.*” Indeed, going beyond appearances to dive into the plants’ intimate functioning requires seeing the plant through the lenses of a modeling act. These lenses can zoom on a particular plant trait or upscale some observations from a fine scale (organ level, single plant) to population or ecosystem scales. Depending on the research question to be addressed, the models can be of different types, ranging from purely statistical models (including machine and deep learning algorithms) to sophisticated mechanistic models i.e. models built using knowledge about the underlying mechanisms (Baker et al., 2018). But amid this diversity, the modelers’ objectives are the same: guide the observation by defining which variables to measure and design the experimental protocols, exhibit and explain the reported correlations, predict future trends under unobserved scenarios, optimize plant management to provide decision support.

Our special topic is representative of this genericity of objectives while covering a wide diversity of model types, applications, and scales. It thus nicely covers several current research trends in the plant growth modeling community. As illustrated in **Figure 1**, the species considered offer a wide spectrum, including (i) crops: maize (Veenstra et al.), tomato (Florakis et al.), soybean (Wang et al.), sorghum (Raymundo et al.), (ii) trees: apple trees (Zhang et al.), pine (Chen and Wang), larch (Cheng et al.), (iii) ecosystems combining both woody and herbaceous species (Chen et al.). The scales range from single plant (Zhang et al., Florakis et al., Chen and Wang) to homogeneous (Veenstra et al., Raymundo et al., Cheng et al.) or mixed multi-species populations (Wang et al., Chen et al.).

**Figure 1:**
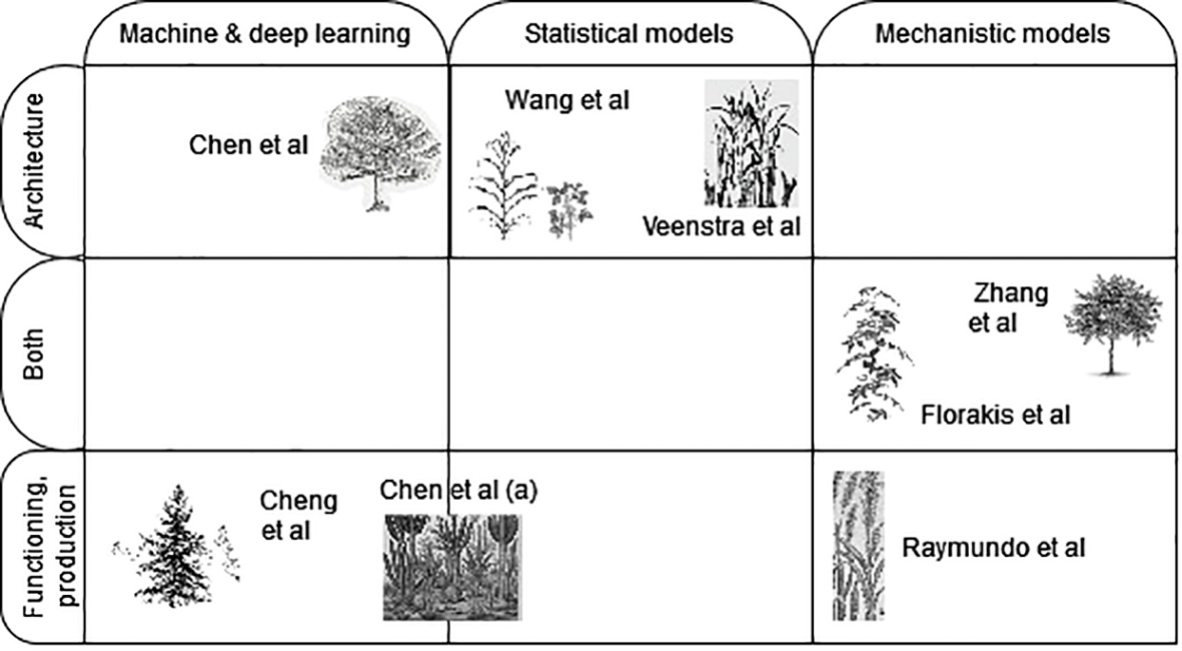
Graphical abstract of the Research Topic. Contributions are positioned with respect to (i) the type of models they present, classified as empirical models (subdivided into machine & deep learning, and statistical models) or mechanistic models, and (ii) the applicative focus being more on the plant architecture, functioning or both. Symbols are representative of the target species considered and of the spatial scale (plant, population, ecosystem).

Regarding model scopes, our topic gathers contributions focusing on either architectural or functional aspects of plant growth, or both (**Figure 1**). Architectural analyses involve the characterization of plant morphology and structure (Bucksch et al., 2017), providing insights into processes such as tillering in maize and crown shape in tree species. Veenstra et al. tested 15 variable combinations, potential predictors of tiller density for Maize. Using a generalized additive model, they proposed a simplified E × M model, where the key management factor identified was plant density, and the environmental factors were temperature and soil fertility-related (NO3 and P) variables. They showed that while point predictions were relatively good, wide prediction intervals highlighted the variability of tiller expression and, consequently, the limited forecast ability of the model. Chen and Wang compared different deep learning and ensemble models to predict the crown radius at a given height from 28 features, including other descriptors of tree structure and their polynomial combinations. They found that an ensemble model combined from Vanila LSTM and LighGBM performed best on their test set and presented the associated SHAP values to assess the respective importance of each descriptor.

Functional trait exploration focuses on the physiological mechanisms underlying plant productivity, including water use efficiency, photosynthesis, and nutrient cycling. Raymundo et al. demonstrated, using the APSIM process-based model (Holzworth et al., 2014), that breeding for limited transpiration in sorghum could increase yield by around 8% on average, with a more pronounced increase in drought-prone regions. Chen et al. explored how the spatial distributions of C, N, and P cycles are determined by the local vegetation characteristics in a desert ecosystem, using both linear models (partial least squares regression) and machine or deep learning (Radom forest, BP neural network). Using the PCA-based Norm value, they selected a minimal data set of community-weighted means among 16 plant traits collected over 300 plots (10mx10m) for, respectively, woody and herbaceous plants. They found that a random forest model combined with kriging-based spatial interpolation of unsampled auxiliary variables performed best. Cheng et al. trained a random forest model to predict the site index of Larch stands (defined as the average dominant tree height at a given base age) under current and future climate scenarios based on climatic, topographic, and soil data. Their model can help anticipate how climate change will affect forest productivity.

Finally, three contributions combine the architectural and functional sides. Wang et al. investigated the factors influencing shading-induced soybean stem lodging in a maize-soybean strip intercropping system: such lodging provokes a substantial decline in yield, a concern for the growers. They fitted logistic and beta law models to follow-up data of the compartment dry weights and their composition in sucrose, lignin, and cellulose, and they analyzed how density affected the model parameters. They found that the shading stress reduced the carbohydrate accumulation rate and duration and suppressed the transport of sucrose from leaves to stem in soybeans, thus resulting in reduced accumulation of lignin and cellulose in the stem and an imbalance in dry matter allocation among soybean organs, leading to stem lodging. Florakis et al. aimed to predict the water consumption of tomato plants grown in a hydroponic greenhouse by exploiting the physiological links between water use efficiency and plant photosynthesis. They analyzed the identifiability of the GreenLab model (Wang et al., 2024) in such a setting and proposed several versions of a simplified stochastic model of light interception. Zhang et al. investigated the impact of scion × interstock combinations on leaf photosynthetic traits and water use efficiency in two apple cultivars grafted onto vigorous or dwarf rootstocks. The 3D architecture-based RATP model allowed simulating virtual scenarios, thus disentangling the interactions between genetic traits and management practices.

These modeling studies call for further work in assembling different models into unified frameworks and more universal simulators to tackle challenging applications in plant breeding, plant adaptability, optimization, and control of crop itineraries.

## Author contributions

VL: Writing – original draft. MK: Writing – review & editing. PR: Writing – review & editing.
